# Dynamic generation of multi-qubit entanglement in the ultrastrong-coupling regime

**DOI:** 10.1038/s41598-019-39265-4

**Published:** 2019-02-27

**Authors:** Xin Liu, Qinghong Liao, Guangyu Fang, Shutian Liu

**Affiliations:** 1 School of Physics and Technology, University of Jinan, Jinan, 250022 People’s Republic of China; 2 Department of Electronic Information Engineering, Nanchang University, Nanchang, 330031 People’s Republic of China; 3 Department of Physics, Harbin Institute of Technology, Harbin, 150001 People’s Republic of China

## Abstract

We propose a dynamic evolution protocol for generating multi-qubit GHZ states in the ultrastrong-coupling regime of circuit QED. By varying the time length of sequences, the protocol works for any coupling strength *g*/*ω*_*r*_ ≥ 0.25. The time for generating the GHZ states in our protocol can be in the subnanoseconds. By taking into account realistic parameters of circuit QED, the degeneracy of fidelity due to decoherence can be as low as 0.02%.

## Introduction

The realization of a controllable platform consisting of two-level systems interacting with a discrete electromagnetic field has been a milestone in the history of quantum physics. Nowadays, the well-known cavity quantum electrodynamics has been greatly studied in the past decades and a lot of applications in quantum information processing have been proposed^1–14^. As usual, all these studies were concentrated on the weak or strong coupling regime where the coupling strength between the two-level system and the cavity mode is much smaller than the frequency of the cavity mode. In recent years, the so-called ultrastrong-coupling regime of a two-level system interacting with a cavity mode was realized experimentally^15–17^, where the coupling strength is comparable to the frequency of the cavity mode, facilitating a concrete realization of the quantum Rabi model. Furthermore, even a deep strong coupling regime where the coupling strength is larger than the frequency of the cavity mode was realized^18^. In this new light-matter interacting regime, a lot of novel phenomena and processes emerged, as conservation of the eigenstate parity^19^, degeneracy of vacuum^20^, absence of Berry phase^21^, non-classical radiation from the thermal cavities^22^, and many efforts were devoted to these phenomena^23–36^. These findings enriched the contents of cavity QED theory, but ideas of how to control and exploit these processes in quantum information processing are still limited^37–42^. In 2012, Romero *et al*. proposed an architecture and a scheme to realize ultrafast logical gates based on the quantum Rabi model^37^. Wu *et al*. proposed to generate Dicke states utilizing selective resonant interactions in the ultrastrong-coupling regime, but the state fidelities were limited^38^. A protocol for harvesting the entanglement of the ground states manifold in the deep strong coupling regime was designed based on the adiabatic processes^39^. However, applications of the ultrastrong-coupling regime are still on the early stage compared with vast schemes in the literature lying in the weak or strong coupling regime.

On the other hand, entanglement plays an important role both in the principles of quantum mechanics and in the implementations of quantum information processing^43,44^. Two-qubit or multi-qubit entanglement is an essential resource in the quantum information tasks. Many efforts have been devoted to the protocols for generating multi-qubit entanglement^5,11–13,45–50^. Unfortunately, due to the fragile nature of quantum entanglement, generation of multi-qubit entanglement has always been a challenging issue. As an expected situation, there are few schemes for generating multi-qubit entanglement in this new ultrastrong-coupling regime and potential applications need to be exploited.

In this paper, we propose a dynamic evolution protocol for generation of GHZ states in the ultrastrong-coupling regime of circuit QED. The scheme works for any coupling strength *g*/*ω*_*r*_ ≥ 0.25. Using a sequence of different time length pulses in different coupling strengths, the multi-qubit entanglement can be generated in subnanoseconds. The advance of this scheme is the quite low decoherence influence due to the special decay processes in the ultrastrong-coupling regime. The influence of inhomogeneous parameters is also discussed, which shows fidelities above 99% of the aimed states under imperfect parameters.

## The Model and Protocol

**Figure 1:**
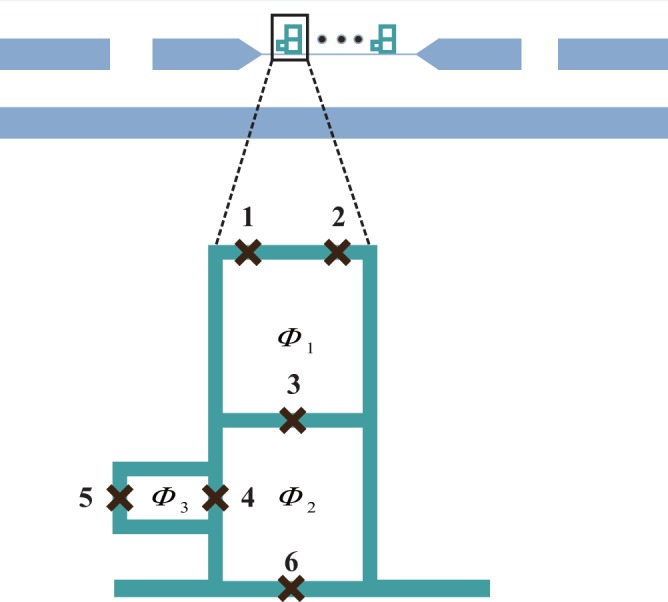
Schematic diagram of the system consisting of six-junction flux qubits galvanically coupled to a transmission-line resonator. The black crosses denote the Josephson junctions and Φ_1_, Φ_2_ and Φ_3_ are external tunable fluxes.

The architecture we consider is schematically illustrated in Fig. 1, where multiple six-junction superconducting flux qubits are galvanically coupled to a coplanar wave-guide resonator. In this qubit design, the longitudinal and transversal coupling of qubits with resonator mode could be tuned by the flux Φ_1_ in the qubit loop^37^ and more coupled qubits could be added by prolonging the length of resonator. By adjusting the coupling with only the longitudinal component and assuming a uniform coupling strength for all the qubits (fluctuation of the coupling strength will be considered later), we obtain the system Hamiltonian to be

1 $$H={\omega }_{r}{a}^{\dagger }a+{\sum }_{i}\frac{{\omega }_{q}}{2}{\sigma }_{x}^{i}+{\sum }_{i}g(a+{a}^{\dagger }){\sigma }_{x}^{i}$$

where *ω*_*r*_ represents the frequency of resonator mode, *ω*_*q*_ is the frequency of a six-junction qubit, and *g* is the coupling strength between the qubits and the resonator mode. The coupling strength *g* can be adjusted by the flux Φ_3_ in the additional loop of a qubit, and each qubit and the flux in each loop of the qubit can be addressed and tuned individually^37^.

In the interaction picture, the Hamiltonian in Eq. (1) takes a form

2 $${H}_{I}=g(a{e}^{-i{\omega }_{r}t}+{a}^{\dagger }{e}^{i{\omega }_{r}t}){S}_{x}$$

with ${S}_{x}=\sum _{i}{\sigma }_{x}^{i}$. The time evolution operator corresponding to the Hamiltonian in Eq. (2) can be written in a factorized way as^7,47,51^

3 $${U}_{I}(t)={e}^{-iA(t){S}_{x}^{2}}{e}^{-iF(t){S}_{x}a}{e}^{-iG(t){S}_{x}{a}^{\dagger }}$$

By substituting the expression of time evolution operator in Eq. (3) into the equation $i[\partial /\partial t{U}_{I}(t)]{U}_{I}^{-1}(t)={H}_{I}(t)$, the coefficients in Eq. (3) can be obtained as

4 $$A(t)=-\,\frac{{g}^{2}}{{\omega }_{r}}[t-\frac{1}{i{\omega }_{r}}({e}^{i{\omega }_{r}t}-1)]$$

5 $$F(t)=\frac{ig}{{\omega }_{r}}({e}^{-i{\omega }_{r}t}-1)$$

6 $$G(t)=\frac{g}{i{\omega }_{r}}({e}^{i{\omega }_{r}t}-1)$$

Therefore, the time evolution operator in the Schrödinger picture is

7 $$U(t)={e}^{-i{\omega }_{r}t{a}^{\dagger }a}{e}^{-i{\omega }_{q}t{S}_{x}/2}{e}^{-iA(t){S}_{x}^{2}}{e}^{-iF(t){S}_{x}a}{e}^{-iG(t){S}_{x}{a}^{\dagger }}$$

In the following, we will mainly use the time evolution operator in Eq. (7) to construct an evolution to GHZ states.

Our protocol for generating GHZ states is implemented in the following four steps. First, tune the coupling strength between the qubits and the resonator to a value *g* and evolve for a time interval *ω*_*r*_*t*_1_ ∈ [0, *π*]. The second step, tune the coupling strength between the qubits and the resonator to a negative value −*g* and evolve for another period *ω*_*r*_*t*_2_ = *π* − *ω*_*r*_*t*_1_. The third step, repeat the step 1 and the fourth step, repeat the step 2.

The total time evolution operator of four steps is written as (see Methods for detailed calculation)

8 $$U={U}_{4}({t}_{2}){U}_{3}({t}_{1}){U}_{2}({t}_{2}){U}_{1}({t}_{1})={e}^{[2i\pi \frac{{g}^{2}}{{\omega }_{r}^{2}}-8i\frac{{g}^{2}}{{\omega }_{r}^{2}}\sin ({\omega }_{r}{t}_{1})]{S}_{x}^{2}}$$

In the total time evolution operator in Eq. (8), the evolutions of the qubits and the resonator are separated and a qubit-qubit XX type interaction is generated despite of individual qubit rotation. This XX type interaction is suitable for the gate for GHZ states^5^, if we set the phase to satisfy

9 $$2\pi \frac{{g}^{2}}{{\omega }_{r}^{2}}-8\frac{{g}^{2}}{{\omega }_{r}^{2}}\,\sin \,({\omega }_{r}{t}_{1})=(1+4m)\frac{\pi }{8}$$

with *m* being an arbitrary integer.

With this phase in rotation in Eq. (8), stating from the qubits state $\underset{i=1}{\overset{N}{\otimes }}{|-\rangle }^{i}$ with an even number of qubits *N*, where ${|-\rangle }^{i}$ and ${|+\rangle }^{i}$ are the eigenstates of the ${\sigma }_{z}^{i}$ operator of every qubit ${\sigma }_{z}^{i}{|-\rangle }^{i}=\,-\,{|-\rangle }^{i}$, ${\sigma }_{z}^{i}{|+\rangle }^{i}={|+\rangle }^{i}$, a GHZ state $\frac{1}{\sqrt{2}}(\underset{i=1}{\overset{N}{\otimes }}{|-\rangle }^{i}+{e}^{i\pi (N+1)/2}\underset{i=1}{\overset{N}{\otimes }}{|+\rangle }^{i})$ is obtained in any qubit-resonator coupling strength *g*/*ω*_*r*_ ≥ 0.25 in the ultrastrong-coupling regime. For an odd number of qubits, an additional single-qubit rotation $\exp (-\,\pi {S}_{x}/4)$ is needed despite of the phase in Eq. (9), and the obtained GHZ state is $\frac{1}{\sqrt{2}}(\underset{i=1}{\overset{N}{\otimes }}{|-\rangle }^{i}+{e}^{i\pi N/2}\underset{i=1}{\overset{N}{\otimes }}{|+\rangle }^{i})$.

## Results

In this section, we simulate the scheme presented above in a four-qubit case. In a four-qubit case, the initial state is ${|-\rangle }^{1}{|-\rangle }^{2}{|-\rangle }^{3}{|-\rangle }^{4}$. This state can be realized by biasing the qubits far away from the degeneracy point with the coupling between the qubits and the resonator shut down, relaxing the qubits to their ground states, and then biasing them back non-adiabatically and applying a π/2 pulse for all the qubits.

**Figure 2:**
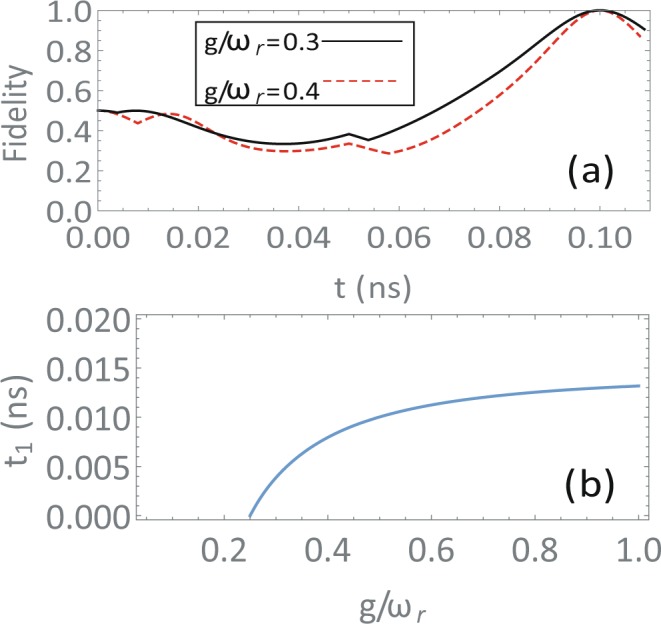
(**a**) The time evolution of the fidelities of the qubits states and the GHZ state for different coupling strengths *g*/*ω*_*r*_ = 0.3 (black solid line) and *g*/*ω*_*r*_ = 0.4 (red dashed line). The unity fidelity is reached at *t* = 0.1 ns under unitary evolution for the four-step protocol. (**b**) The needed time of the first step *t*_1_ under different coupling strengths (scaled by the resonator frequency *ω*_*r*_). By varying the time of the first step *t*_1_, the protocol works for any coupling strength *g*/*ω*_*r*_ ≥ 0.25.

By setting the initial resonator state in the vacuum state $|0\rangle$, the evolution of fidelity of the qubits state and the GHZ state is presented in Fig. 2(a) for coupling strengths *g*/*ω*_*r*_ = 0.3 and *g*/*ω*_*r*_ = 0.4. The frequency of the first mode of a transmission line resonator could be 1 × 2*π* ∼ 10 × 2*π* GHz. In the simulation, a frequency of 10 × 2*π* GHz is chosen. It could be seen that in *t* = 2*π*/*ω*_*r*_ = 0.1 ns exact GHZ states are obtained for any coupling strength. In Fig. 2(b), the evolution time of the first step is presented according to Eq. (9). It could be seen that the evolution time *t*_1_ needed grows slightly with the increase of qubit-resonator coupling strength, and evolutions to GHZ states in different coupling strengths could be realized by adjusting the evolution time *t*_1_.

In the last paragraph, we simulate our protocol with a unitary evolution, and a unity fidelity can be obtained. However, the advance of the protocol will be prominent only when one takes into account the decay of the procedure. In the ultrastrong-coupling regime, the standard master equation fails to describe the decoherence of system because it predicts some unphysical results. A proper way is to expand the system-bath interaction Hamiltonian in the basis of system Hamiltonian eigenstates and after applying the standard Markov approximation and tracing out the reservoirs degrees of freedom, a master equation describing the decoherence of quantum Rabi model can be obtained as^22,52^

10 $$\dot{\rho }(t)=i[\rho (t),H]+{\sum }_{\lambda }{\sum }_{j,k > j}{{\rm{\Gamma }}}_{\lambda }^{jk}D[|j\rangle \langle k|]\rho (t)$$

where $|j\rangle$ is the j-th eigenstate of the system Hamiltonian with the eigenstates labeled by the corresponding eigenvalues in an increasing order and the super-operator $D[O]\rho$ takes the form $D[O]\rho =\frac{1}{2}(2O\rho {O}^{\dagger }-\rho {O}^{\dagger }O-{O}^{\dagger }O\rho )$. The subscript *λ* represent the decay from the cavity (*λ*_*c*_) and the decay from the qubits (*λ*_1_, *λ*_2_, *λ*_3_, *λ*_4_). The relaxation coefficients can take a simplified form ${{\rm{\Gamma }}}_{\lambda }^{jk}={\gamma }_{\lambda }{|{C}_{j,k}^{(\lambda )}|}^{2}$ with *γ*_*λ*_ being the standard damping rates in a weak coupling scenario and ${C}_{jk}^{(\lambda )}=\langle j|({c}_{\lambda }+{c}_{\lambda }^{\dagger })|k\rangle$, after assuming constant spectral densities and constant system-bath coupling strengths.

**Figure 3:**
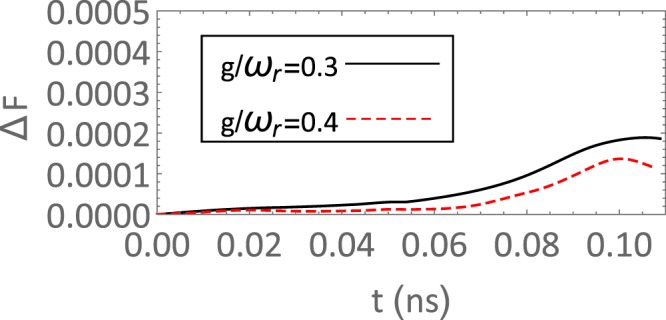
The evolution of fidelity deviations (Δ*F* = *F* − *F*_*d*_ with *F* being the fidelity under the unitary evolution and *F*_*d*_ being the fidelity with decay) with time for different coupling strengths *g*/*ω*_*r*_ = 0.3 (black solid line) and *g*/*ω*_*r*_ = 0.4 (red dashed line). The other parameters are *ω*_*r*_ = 10 × 2*π* GHz, *Q* = 2 × 10^5^, *T*_1_ = 1.5 *μ*s.

In the simulation, a qubit relaxation time *T*_1_ = 1.5 μs and a cavity quality factor *Q* = 2 × 10^5^ are assumed, which has been realized in a flux qubit experiment^53^ and is not very high for a transmission line resonator^54^. In Fig. 3, fidelity deviations Δ*F* between the unitary evolutions and the evolutions with the cavity and qubits decay are plotted. It is shown that the deviations grow as time passes and fidelity deviations under 0.02% can be obtained at *t* = 0.1 ns, which is greatly suppressed compared with some schemes working in the strong coupling regime^12,46,50^. In the above master equation, dephasing effect was not included in calculation. However, by considering a dephasing time of more than 1 μs in ref.^55^, we can still hope a state fidelity up to 99%. Or in an alternative way, like the method done in ref.^18^, tuning the flux qubit in the degeneracy point, and setting the frequency of resonator much larger than the qubit gap Δ will also consist the model of Eq. (2) used in our scheme, while dephasing effect will be largely suppressed.

**Figure 4:**
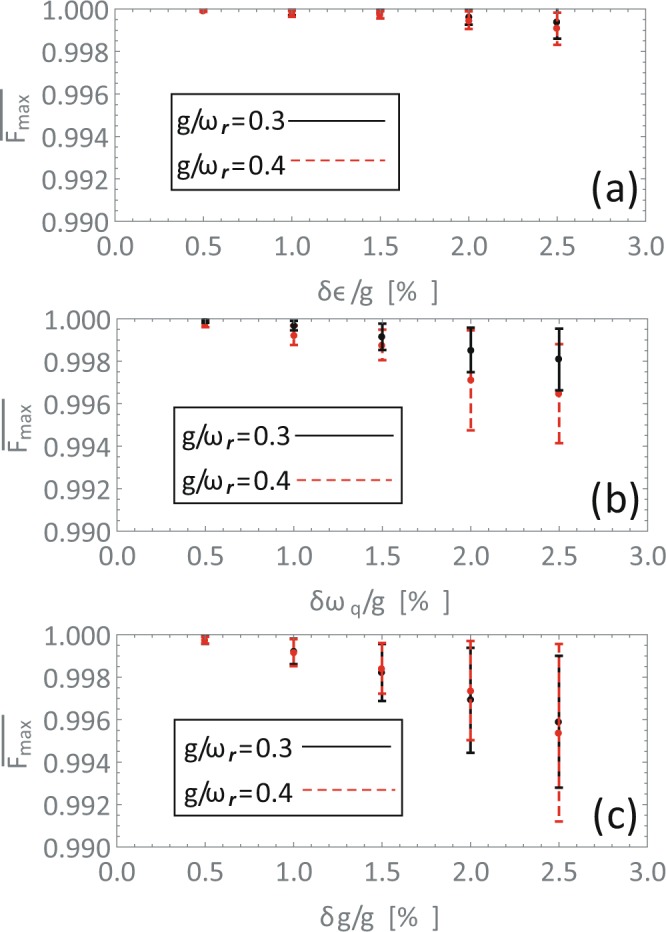
The influences on the fidelities caused by inhomogeneous (**a**) *ε*, (**b**) *ω*_*q*_ and (**c**) g (corresponding to the strengths of the *σ*_*z*_, *σ*_*x*_ components of the qubits and the coupling strength of the qubits and the resonator) individually. The longitudinal axes show the mean fidelities and the standard deviations of the fidelities for a fixed deviation of the parameter (scaled by the coupling strength *g*). The parameters are *ω*_*r*_ = 10 × 2*π* GHz, *g*/*ω*_*r*_ = 0.3 (black solid line) and *g*/*ω*_*r*_ = 0.4 (red dashed line) and the number of the random evolutions is *n*_*r*_ = 100.

For a realistic experimental implementation of circuit QED system, the fluctuations of parameters of qubits and qubit-resonator coupling strengths are unavoidable. We simulate the influence of fluctuations of these parameters on the fidelities of aimed states in Fig. 4. We evaluate mean fidelities and standard deviations of the maximal fidelities in a few single evolution procedures with random values of a parameter. We show the influence of these fluctuations of parameters with fluctuation of one parameter in one figure. For one parameter, we assumed a random value for each of the qubit in Gaussian distribution with a standard deviation *δr*. For each deviation, 100 times single evolution of the protocol is calculated and the mean value and deviation of the maximal fidelities are calculated. The influences of random *δε*, *δω*_*q*_ and *δg* (corresponding to the strengths of the *σ*_*z*_, *σ*_*x*_ components of the qubits and the coupling strength of the qubits and the resonator, respectively) are shown in Fig. 4(a–c) respectively, which rise from fluctuations of the magnetic flux and fabrication imperfections. It is shown that the mean fidelities decrease and the corresponding standard deviations increase as growing of the fluctuations of parameters, as expected. Under the fluctuations of parameters with standard deviation 2.5%, the fidelities of protocol still keep up to 99% and the fluctuation of coupling strength *g* changes the fidelity more dramatically.

## Discussion

In practical implementation of our scheme in circuit QED, the most challenging problem may be the control pulses with high switching frequency and well-defined shape. Indeed, high switching frequency pulses up to 10 GHz have already been realized in experiment^56^. We can expect pulses with higher frequency will be realized in state-of-the-art circuit QED experiments. Besides, with the same parameters in Fig. 2a, we simulated the fidelity evolution with coupling strength *g* in a smooth error function profile as what were did in refs^57,58^ with a standard deviation 0.005 ns of Gaussian and a maximal fidelity above 99.1% can be obtained. The coplanar transmission line resonator is a one-dimension cavity with multiple modes. Without the rotating-wave approximation, higher modes will have influential couplings with the qubits. Based on the multi-mode quantum Rabi model, the higher modes don’t change the form of time evolution operator in Eq. (7), so they won’t change the fidelity of the protocol. The only effect is the summary of multiple modes in the exponential component in Eq. (7) will modify the phases in Eq. (18), resulting in a new phase equation which time *t*_1_ should satisfy ${\sum }_{\tilde{n}=1}^{{n}_{c}}2\pi ({g}_{\tilde{n}}^{2}/{\omega }_{\tilde{n}}^{2})-8({g}_{\tilde{n}}^{2}/{\omega }_{\tilde{n}}^{2})\sin ({\omega }_{\tilde{n}}{t}_{1})=\pi /8$, where the *g*_*ñ*_ and *ω*_*ñ*_ are coupling strength and mode frequency of mode *ñ* and *n*_*c*_ is the cutoff number of cavity modes. With this time length of *t*_1_ satisfying new phase equation, the protocol could be implemented with unity fidelity. It will be noticed that there already exist some schemes for generating multi-qubit entanglement in the ultrastrong-coupling regime^29,38,39,47,59,60^, which may have some similarities with the protocol here, especially the scheme in ref.^47^. However, we clarify the protocol here is exactly an extension of scheme in ref.^47^. In Fig. 2b, it will be find our protocol reduce to the protocol in ref.^47^ at *g*/*ω*_*r*_ = 0.25, where the time *t*_1_ reduces to zero and the one-step protocol in ref.^47^ is recovered. On the other hand, compared with the protocol in ref.^47^ working for some special coupling strengths fixed by two integers, our protocol works for any coupling strength by varying the time length of first step, releasing a parameter constriction.

In summary, we propose a dynamic scheme for generating multi-qubit GHZ states in the ultrastrong-coupling regime. The scheme can generate the GHZ states with unity fidelity in subnanoseconds under unitary evolution. More astonishingly, the deviation caused by the decay of the qubits and resonator is greatly suppressed to below 0.02%. The scheme works for any qubit-resonator coupling strength *g*/*ω*_*r*_ ≥ 0.25 and the operation time does not change with the increasing of the qubit number. The influences of inhomogeneous parameters are also discussed. We hope the scheme can provide a realizable method for generating multi-qubit entanglement in the ultrastrong-coupling regime of cavity QED.

## Methods

The time evolution operator in Eq. (7) corresponding to step one and step three can be rewritten as

11 $$\begin{array}{rcl}{U}_{1,3} & = & {e}^{-i{\omega }_{r}t{a}^{\dagger }a}{e}^{-i{\omega }_{q}t{S}_{x}/2}{e}^{-iA(t){S}_{x}^{2}}{e}^{-\frac{1}{2}{|F(t)|}^{2}{S}_{x}^{2}}{e}^{[-iF(t){S}_{x}a-i{F}^{\ast }(t){S}_{x}{a}^{\dagger }]}\\  & = & {e}^{-i{\omega }_{r}t{a}^{\dagger }a}{e}^{-i{\omega }_{q}t{S}_{x}/2}{e}^{[-iA(t)-\frac{1}{2}{|F(t)|}^{2}]{S}_{x}^{2}}{\rm{D}}(\,-\,i{F}^{\ast }(t){S}_{x})\end{array}$$

with ${\rm{D}}(\beta {S}_{x})=\exp [(\beta {a}^{\dagger }-{\beta }^{\ast }a){S}_{x}]$ being the controlled coherent displacement of the field. In step two and step four, the system Hamiltonian in the interaction picture can be written as

12 $${H}_{I}=-\,g(a{e}^{-i{\omega }_{r}t}+{a}^{\dagger }{e}^{i{\omega }_{r}t}){S}_{x}$$

The corresponding time evolution operator in the Schrödinger picture is

13 $$U(t)={e}^{-i{\omega }_{r}t{a}^{\dagger }a}{e}^{-i{\omega }_{q}t{S}_{x}/2}{e}^{-iA^{\prime} (t){S}_{x}^{2}}{e}^{-iG^{\prime} (t){S}_{x}{a}^{\dagger }}{e}^{-iF^{\prime} (t){S}_{x}a}$$

with

14 $$A^{\prime} (t)=(\frac{{g}^{2}}{{\omega }_{r}})[\frac{1}{-i{\omega }_{r}}({e}^{-i{\omega }_{r}t}-1)-t]$$

15 $$G^{\prime} (t)=-\,\frac{g}{i{\omega }_{r}}({e}^{i{\omega }_{r}t}-1)$$

16 $$F^{\prime} (t)=\frac{g}{i{\omega }_{r}}({e}^{-i{\omega }_{r}t}-1)$$

The time evolution operator corresponding to step two and step four can be rewritten as

17 $$\begin{array}{rcl}{U}_{2,4} & = & {e}^{-i{\omega }_{r}t{a}^{\dagger }a}{e}^{-i{\omega }_{q}t{S}_{x}/2}{e}^{-iA^{\prime} (t){S}_{x}^{2}}{e}^{\frac{1}{2}{|F^{\prime} (t)|}^{2}{S}_{x}^{2}}{e}^{[-i{F^{\prime} }^{\ast }(t){S}_{x}{a}^{\dagger }-iF(t){S}_{x}a]}\\  & = & {e}^{-i{\omega }_{r}t{a}^{\dagger }a}{e}^{-i{\omega }_{q}t{S}_{x}/2}{e}^{[-iA^{\prime} (t)+\frac{1}{2}{|F^{\prime} (t)|}^{2}]{S}_{x}^{2}}{\rm{D}}(\,-i{F^{\prime} }^{\ast }(t){S}_{x})\end{array}$$

The total time evolution operator of the four steps will be

18 $$\begin{array}{rcl}U & = & {U}_{4}({t}_{2}){U}_{3}({t}_{1}){U}_{2}({t}_{2}){U}_{1}({t}_{1})\\  & = & {e}^{-i{\omega }_{r}{t}_{2}{a}^{\dagger }a}{e}^{[-iA^{\prime} ({t}_{2})+\frac{1}{2}{|F^{\prime} ({t}_{2})|}^{2}]{S}_{x}^{2}}{\rm{D}}(\,-i{F}^{^{\prime} * }({t}_{2}){S}_{x}){e}^{-i{\omega }_{r}{t}_{1}{a}^{\dagger }a}{e}^{[-iA({t}_{1})-\frac{1}{2}{|F({t}_{1})|}^{2}]{S}_{x}^{2}}{\rm{D}}(\,-i{F}^{* }({t}_{1}){S}_{x})\\  &  & {e}^{-i{\omega }_{r}{t}_{2}{a}^{\dagger }a}{e}^{[-iA^{\prime} ({t}_{2})+\frac{1}{2}{|F^{\prime} ({t}_{2})|}^{2}]{S}_{x}^{2}}{\rm{D}}(\,-\,i{F}^{^{\prime} * }({t}_{2}){S}_{x}){e}^{-i{\omega }_{r}{t}_{1}{a}^{\dagger }a}{e}^{[-iA({t}_{1})-\frac{1}{2}{|F({t}_{1})|}^{2}]{S}_{x}^{2}}{\rm{D}}(\,-\,i{F}^{* }({t}_{1}){S}_{x})\\  & = & {e}^{-i\pi {a}^{\dagger }a}{e}^{[-iA^{\prime} ({t}_{2})+\frac{1}{2}{|F^{\prime} ({t}_{2})|}^{2}]{S}_{x}^{2}}{\rm{D}}(\,-i{F}^{^{\prime} * }({t}_{2}){e}^{i{\omega }_{r}{t}_{1}}{S}_{x}){e}^{[-iA({t}_{1})-\frac{1}{2}{|F({t}_{1})|}^{2}]{S}_{x}^{2}}{\rm{D}}(\,-\,i{F}^{* }({t}_{1}){S}_{x})\\  &  & {e}^{-i\pi {a}^{\dagger }a}{e}^{[-iA^{\prime} {t}_{2})+\frac{1}{2}{|F^{\prime} ({t}_{2})|}^{2}]{S}_{x}^{2}}{\rm{D}}(\,-\,i{F}^{^{\prime} * }({t}_{2}){e}^{i{\omega }_{r}{t}_{1}}{S}_{x}){e}^{[-iA({t}_{1})-\frac{1}{2}{|F({t}_{1})|}^{2}]{S}_{x}^{2}}{\rm{D}}(\,-\,i{F}^{* }({t}_{1}){S}_{x})\\  & = & {e}^{[-2iA^{\prime} ({t}_{2})+{|F^{\prime} ({t}_{2})|}^{2}-2iA({t}_{1})-{|F({t}_{1})|}^{2}]{S}_{x}^{2}}{e}^{i{\rm{Im}}[-i{F}^{^{\prime} * }({t}_{2}){e}^{i{\omega }_{r}{t}_{1}}iF({t}_{1}){S}_{x}^{2}]}{\rm{D}}[(\,-\,i{F}^{^{\prime} * }({t}_{2}){e}^{i{\omega }_{r}{t}_{1}}-i{F}^{* }({t}_{1})){e}^{-i\pi }{S}_{x}]\\  &  & {e}^{i{\rm{Im}}[-i{F}^{^{\prime} * }({t}_{2}){e}^{i{\omega }_{r}{t}_{1}}iF({t}_{1}){S}_{x}^{2}]}{\rm{D}}[(\,-\,i{F}^{^{\prime} * }({t}_{2}){e}^{i{\omega }_{r}{t}_{1}}-i{F}^{* }({t}_{1})){S}_{x}]\\  & = & {e}^{[-2iA^{\prime} ({t}_{2})+{|F^{\prime} ({t}_{2})|}^{2}-2iA({t}_{1})-{|F({t}_{1})|}^{2}]{S}_{x}^{2}}{e}^{i2{\rm{Im}}[-i{F}^{^{\prime} * }({t}_{2}){e}^{i{\omega }_{r}{t}_{1}}iF({t}_{1}){S}_{x}^{2}]}\\  &  & {e}^{i{\rm{Im}}[(-i{F}^{^{\prime} * }({t}_{2}){e}^{i{\omega }_{r}{t}_{1}}-i{F}^{* }({t}_{1})){e}^{-i\pi }(iF^{\prime} ({t}_{2}){e}^{-i{\omega }_{r}{t}_{1}}+iF({t}_{1})){S}_{x}^{2}]}\\  & = & \exp \{[2i\pi \frac{{g}^{2}}{{\omega }_{r}^{2}}-4i\frac{{g}^{2}}{{\omega }_{r}^{2}}\,\sin ({\omega }_{r}{t}_{1})]{S}_{x}^{2}\}\exp \{[-4i\frac{{g}^{2}}{{\omega }_{r}^{2}}\,\sin ({\omega }_{r}{t}_{1})]{S}_{x}^{2}\}\\  & = & {e}^{[2i\pi \frac{{g}^{2}}{{\omega }_{r}^{2}}-8i\frac{{g}^{2}}{{\omega }_{r}^{2}}\sin ({\omega }_{r}{t}_{1})]{S}_{x}^{2}}\end{array}$$

where we have omitted rotation rising from *S*_*x*_ component which just generates trivial single qubit rotation. In the calculation, equations ${\rm{D}}(\alpha ){\rm{D}}(\beta )={e}^{i{\rm{Im}}(\alpha {\beta }^{\ast })}{\rm{D}}(\alpha +\beta )$ and ${e}^{-i\theta {a}^{\dagger }a}{\rm{D}}(\alpha ){e}^{i\theta {a}^{\dagger }a}={\rm{D}}(\alpha {e}^{-i\theta })$ are used.
